# Media and breastfeeding: Friend or foe?

**DOI:** 10.1186/1746-4358-3-15

**Published:** 2008-08-04

**Authors:** Jane D Brown, Sheila Rose Peuchaud

**Affiliations:** 1 School of Journalism and Mass Communication, University of North Carolina at Chapel Hill, Chapel Hill, North Carolina, USA

## Abstract

The mass media have the potential to be powerful friends or foes in promoting breastfeeding. The media could help by putting the issue of breastfeeding on policy agendas and by framing breastfeeding as healthy and normative for baby and mother. Currently, however, it looks as if the media are more often contributing to perceptions that breastfeeding is difficult for mothers and potentially dangerous for babies. This paper presents a brief overview of research on the media and breastfeeding, some insights into the market forces and human psychological factors that may play into media representations of breastfeeding, and strategies to help breastfeeding advocates work more effectively with the media.

## Debate

### What we know about representations of breastfeeding in the media

Little systematic research has been done on the portrayal or effects of breastfeeding in the media. One analysis of the popular Dr. Spock baby care books found that in the early editions (1945, 1968) Spock promoted breastfeeding as "the natural way" and suggested that mothers would physically benefit from the practice [1]. By the 1990s, however, Spock's advice had shifted to a more medical paradigm, arguing that breastfeeding will provide immunity for the baby and that breast milk is easier for the baby to digest [1].

A recent analysis of 615 articles published between 1997 and 2003 in seven popular parenting, general women's, and African American magazines in the United States found that although the magazines provided more information on breastfeeding than formula feeding, responsibility was placed on the mother [2]. The importance of social policy and partner support were rarely mentioned [2]. Another analysis showed that the number of advertisements promoting infant formula and related products in the influential *Parents *magazine increased dramatically between 1972 and 1999, just as the percentage of women initiating breastfeeding declined [3].

Two alarming characteristics of media representations of breastfeeding are the association of breastfeeding and dead babies, and the asymmetrical treatment of mothers of color. In 1994, the *Wall Street Journal *ran a story depicting white mothers suffering from "insufficient milk syndrome" and their babies as the victims of a medical establishment that encouraged breastfeeding at all costs. In contrast, the news coverage of the 1997 trial of Tabitha Walrond, a young black mother convicted of negligent homicide because her two month old son died from malnutrition, blamed Walrond for failing to live up to her responsibilities as a mother. Despite previous breast reduction surgery, Walrond had not been told that such procedures might result in insufficient breast milk production. As a recipient of public assistance, Walrond had also run into numerous bureaucratic obstacles and was denied care when she sought medical attention for her baby in the weeks before his death. Nonetheless, rather than framing her as a victim of the medical system, as the white women were, the media portrayed Walrond as a criminally negligent mother [4].

When Walrond's case was dramatized a couple of years later on the popular TV show "Chicago Hope," breastfeeding was portrayed as problematic and potentially lethal. Significantly, the parents in the episode were white and middle-class, a demographic with significantly better access to timely medical care. They were subjected, nonetheless, to a criminal investigation (although later exonerated) when their breastfed baby died of malnutrition. The episode also suggested that the Baby Friendly Hospitals Initiative was a coercive contract that forced a parent to breastfeed – even to the point of killing a baby – rather than a set of commitments on the part of the hospital to encourage and enable successful breastfeeding initiation. Even more troubling, the episode was apparently part of a larger campaign by the pharmaceutical industry to tell the public about the "risks associated with breastfeeding" [5].

### The forces that shape media representations of breastfeeding

Although now a decade old, the Walrond and "Chicago Hope" cases illustrate two fundamental facts about U.S. media: (1) the media are increasingly profit-driven conglomerates; (2) they are not in the business of health education. The primary way media increase profits is to attract consumers, because consumers attract advertisers. Advertisers are basically buying the right to sell their products to desirable audiences, so media content is simply the bait to lure the audience. Big advertisers, such as the infant formula companies with large advertising budgets, have a lot of say in this kind of media environment. Breastfeeding advocates, who seldom have the same kind of money to spend on advertising, have less clout. If the content that advocates generate does not conform to basic news or entertainment values or turns media consumers away, it will not be used, and/or it will be distorted.

A recent line of psychological inquiry, known as Terror Management Theory (TMT), may provide more insight into why breastfeeding is so seldom seen in the media, and why public breastfeeding evokes such visceral controversy. According to TMT, people have a deep-seated aversion to reminders of the physical, animal nature of humanity because such reminders raise awareness of our mortality. One study found that thinking about death made people react more negatively to a scenario about women breastfeeding in public and made them avoid a potential task partner who was described as breastfeeding in another room [6]. The negative response apparently was directly related to breastfeeding, because images of women simply posing with babies did not evoke negative feelings [6].

The media may have realized what some research now suggests are deep-seated negative reactions to seeing breastfeeding mothers. This also suggests how difficult it may be to shift cultural norms using media that are increasingly visual, and typically show breasts as erotic rather than functional.

### Strategies for working with media

Despite these challenges, the media could play important and positive roles in promoting breastfeeding. Advocates for other public health issues such as tobacco control have learned a number of lessons about what it would take [7]. Media campaigns are most effective when messages are simple, clear, tailored to the needs and tastes of specific audience segments, and placed in channels that reach important target audiences. Straightforward messages to expectant and new mothers promoting the health effects of breastfeeding are important. But campaigns should also focus on changing cultural norms so that breastfeeding mothers are seen as a natural part of the community and workplace. The news media can be helpful in promoting public policies such as paid maternity leave, baby-friendly work environments, and affordable childcare that will make it easier for working mothers to breastfeed.

Sustained exposure over time is key. One strategy would be to develop partnerships with media producers to assure that the issue of breastfeeding stays on the media news agenda, and that news and entertainment portrayals are positive. Advocates could develop relationships with producers and screenwriters, supplying story lines that make breastfeeding seem healthy, normative, and positive. Such "entertainment-education" efforts on behalf of other health issues, such as unplanned pregnancy and emergency contraception, have been effective in increasing audience awareness [8]. These Hollywood advocates could also serve as sentinels, vigilant about story lines, like the one in "Chicago Hope," that threaten to undermine the desired message so proponents could be ready to protest. Breastfeeding advocates also could provide "information subsidies" to news organizations. Providing packages of content that can be aired with minimal editing, and access to credible, articulate spokespeople and breastfeeding mothers can help ensure that the media paint the desired picture.

## Competing interests

The authors declare that they have no competing interests.

## Authors' contributions

JDB wrote the initial draft in consultation with SP who made substantial conceptual contributions and changes.
